# Reducing Error in ECG Forward Simulations With Improved Source Sampling

**DOI:** 10.3389/fphys.2018.01304

**Published:** 2018-09-21

**Authors:** Jess Tate, Karli Gillette, Brett Burton, Wilson Good, Brian Zenger, Jaume Coll-Font, Dana Brooks, Rob MacLeod

**Affiliations:** ^1^Department of Bioengineering, University of Utah, Salt Lake City, UT, United States; ^2^Scientific Computing and Imaging Institute, University of Utah, Salt Lake City, UT, United States; ^3^Institute of Biophysics, Medical University of Graz, Graz, Austria; ^4^Computational Radiology Lab, Children's Hospital, Boston, MA, United States; ^5^SPIRAL Group, Department of Electrical and Computer Engineering, Northeastern University, Boston, MA, United States

**Keywords:** ECG imaging, ECG forward simulation, cardiac source sampling, epicardial potentials, body-surface potentials

## Abstract

A continuing challenge in validating electrocardiographic imaging (ECGI) is the persistent error in the associated forward problem observed in experimental studies. One possible cause of this error is insufficient representation of the cardiac sources; cardiac source measurements often sample only the ventricular epicardium, ignoring the endocardium and the atria. We hypothesize that measurements that completely cover the pericardial surface are required for accurate forward solutions. In this study, we used simulated and measured cardiac potentials to test the effect of different levels of spatial source sampling on the forward simulation. Not surprisingly, increasing the source sampling over the atria reduced the average error of the forward simulations, but some sampling strategies were more effective than others. Uniform and random distributions of samples across the atrial surface were the most efficient strategies in terms of lowest error with the fewest sampling locations, whereas “single direction” strategies, *i.e*., adding to the atrioventricular (AV) plane or atrial roof only, were the least efficient. Complete sampling of the atria is needed to eliminate errors from missing cardiac sources, but while high density sampling that covers the entire atria yields the best results, adding as few as 11 electrodes on the atria can significantly reduce these errors. Future validation studies of the ECG forward simulations should use a cardiac source sampling that takes these considerations into account, which will, in turn, improve validation and understanding of ECGI.

## 1. Introduction

Electrocardiographic Imaging (ECGI) is a promising technology for diagnosing and treating cardiac arrhythmias (Pullan et al., 2010; Rudy and Lindsay, 2015). Its goal is to compute some formulation of cardiac sources from known patient torso geometry (typically extracted from medical imaging) and body-surface potential mapping (BSPM) recordings (Barr et al., 1977; Plonsey and Barr, 1987; Plonsey and van Oosterom, 1991; Gulrajani, 1998). This computation is possible by first establishing a model of the ECG from knowledge of cardiac sources and geometry, known as a numerical forward simulation (MacLeod and Buist, 2010) and then inverting this process to solve the associated inverse problem (Pullan et al., 2010). Establishing well-validated ECG forward simulations is, therefore, critical to developing ECGI as a technology.

The purpose of an ECG forward simulation is to predict the electric potential response through a passive volume conductor, i.e., the torso, from cardiac sources (MacLeod and Buist, 2010). Cardiac sources are represented in the literature in several ways, but the most common and most readily measured method is a surface of potentials surrounding the myocardium (Barr et al., 1977; Messinger-Rapport and Rudy, 1986; Plonsey and Barr, 1987; Plonsey and van Oosterom, 1991; Gulrajani, 1998). Predicting the resulting ECGs requires solving a partial differential equation using numerical techniques, such as boundary or finite element methods (BEM and FEM, respectively) (Johnson et al., 1993; Johnson, 1997, 2015; MacLeod and Buist, 2010).

Despite the existence of well-established methods of the ECG forward simulation, previous validation studies have consistently shown differences that were higher than might be expected between simulated and measured body-surface potentials, such as higher overall error and changes in extrema location (Ramsey et al., 1977; Bear et al., 2015). The ECG forward problem is well behaved, and we have sufficient confidence in all aspects of the simulation and measurement protocols to expect errors well below those reported. This disparity between confidence in the simulation approaches and persistent errors in experimental validation, along with the sensitivity of ECGI to model errors due to its ill-posed nature (Pullan et al., 2010), provides powerful motivation to explore possible explanations.

One as yet unexplored source of error in these studies is insufficient cardiac source representation, i.e., either inadequate coverage or spatial density of coverage of the cardiac sources. For example, many experimental validation studies use an epicardial sock electrode array to record cardiac surface potentials from the animal heart (Ramsey et al., 1977; Stanley et al., 1986; Shome and MacLeod, 2007; Bear et al., 2015). A common limitation of these epicardial socks is that they position electrodes on the ventricles only, ignoring the atria. Not only does such a set up exclude measurement of atrial sources, but some ventricular sources, such as locations either on the apex or at the base of the heart, lack either adequate spatial coverage or stable mechanical contact by sock electrodes. Such conditions are problematic as the mathematical formulation of the ECG forward simulation with potential sources assumes a complete and closed representative surface that is adequately sampled; the compromises driven by practical limitations in experiments suggest that missing sources exist and they could have a significant impact on the predicted potential values on the torso surface (Barr et al., 1977). Our goal was to examine some aspects of this dilemma, using a combination of experimental and numerical approaches.

In addition to experimental studies, we can also use computer simulation to help answer questions about the effect of cardiac sampling on the forward simulation. Simulation methods such as pseudo-bidomain (Vigmond et al., 2003, 2008) and cellular automaton (Schulze et al., 2015) can predict full pericardial potentials in a way that cannot be measured experimentally due to regions of the epicardium being inaccessible to measurement. Using simulated potentials together with experimentally recorded values provides a more complete evaluation of the effect of pericardial source sampling.

In this study, we tested the impact of cardiac source representation of the atrial region on ECG forward simulations. We hypothesize that, in the context of forward simulations from epicardial potentials, measurements that completely cover the heart are required for accurate prediction of the body-surface potentials. To test this hypothesis, we used simulated and measured cardiac potentials to determine the effect of different levels of sampling on a typical forward simulation pipeline (Burton et al., 2011). Our results support this hypothesis and encourage us to propose some sampling strategies that may minimize error resulting from incomplete sampling of cardiac sources.

## 2. Methods

We analyzed the effect of source representation coverage and density of the atrial region of the heart on ECG forward simulations by sampling the cardiac source with a range of strategies, and then used those sources in our ECG forward simulation pipeline. We tested these sampling strategies on three different geometries and source models: (1) simulated epicardial potentials using the CARP (Vigmond et al., 2003, 2008) cardiac propagation modeling software package, (2) a second set of simulations provided in the EDGAR database (Aras et al., 2015) by the Biomedical Engineering team a the Karlsruhe Institute of Technology, KIT (Schulze et al., 2015), and (3) one experimentally recorded dataset from the CardioVascular Research and Training Institute (CVRTI) at the University of Utah using a unique “cage” electrode (Milanic et al., 2014), also available in the EDGAR database (Aras et al., 2015). We then computed ECG forward simulations from subsampled versions of the original sources, which we compared to FEM simulations from our ground truth cardiac potential sources. We also performed experiments in which we recorded source potentials with a ventricular sock and an electrode plaque placed on the atria and used these recorded potentials in our simulation pipeline to compare the predicted body-surface potentials with and without the additional atrial potential sources.

### 2.1. Datasets

#### 2.1.0.1. CARP dataset

The set of cardiac potentials generated using the CARP (Vigmond et al., 2003, 2008) modeling software consisted of simulated extracellular potentials using the pseudo-bidomain method (Bishop and Plank, 2011) in an isolated rabbit ventricle model previously described (Deo et al., 2009). The four pacing profiles were sinus rhythm, left ventricle (LV) free wall pacing, right ventricle (RV) free wall pacing, and apical pacing. The heart geometry was then manually registered and scaled to a human torso geometry of of dimensions ~ 36 × 22 × 40 cm, 771 nodes, and an internodal distance of 24.6 mm (MacLeod et al., 1995; Shome and MacLeod, 2007; Milanic et al., 2014). An ellipsoidal cap was placed on a mesh of the epicardial surface of the ventricles (to replicate a typical sock array) by fitting a precomputed ellipsoid mesh to the points near the base of the ventricles and clipping it to cover the open region in the sock. The combination of the sock mesh and the ellipsoid cap formed a pericardial mesh of dimensions ~ 6 × 6 × 7 cm with 498 nodes with an average internodal spacing of 5.3 mm. To compute the potentials on both the cap of the mesh and the torso surface, we used the previously computed ventricular surface extracellular potentials from both the endocardial and epicardial surfaces and the FEM approach in SCIRun (http://scirun.org, Parker et al., 1997; MacLeod et al., 2004) with the Forward/Inverse Toolkit (Burton et al., 2011). This calculation consisted of generating a tetrahedral mesh for the region between the heart and torso surface, including the vertex locations for the pericardial mesh with the ellipsoid cap. Then for each time step, the endocardial and epicardial potentials were used to set the Dirichlet boundary conditions along the cardiac surface and Neumann boundary conditions on the torso surface to solve for the potentials distribution throughout the homogeneous torso volume. The potentials were extracted at the torso and pericardial surfaces to use in the subsequent sampling tests described below.

#### 2.1.0.2. KIT dataset

The KIT geometric model of a single heart and torso geometry was generated from a patient scan (Schulze et al., 2015) and is available on the EDGAR database (http://edgar.sci.utah.edu, Aras et al., 2015). The torso surface had the dimensions ~ 47 × 30 × 35 cm, 2002 nodes, and an internodal distance of 19.0 mm. The cardiac potentials computed from this model, also available from EDGAR, consisted of four activation profiles: septal, RV free wall, LV free wall, and apical pacing. In contrast to the pseudo-bidomain approach using CARP, the KIT investigators computed cardiac potentials using a cellular automaton approach for the activation sequence, and calculated first the transmembrane potentials based on the activation times with a monodomain simulation and the ten Tusscher electrophysiological model (ten Tusscher and Panfilov, 2006; Loewe et al., 2015) and then the extracellular potentials using the bidomain approach (Schulze et al., 2015). As in the CARP dataset, we added an ellipsoidal cap on a mesh of the epicardium to form a pericardial mesh of dimensions ~ 13 × 19 × 10 cm with 532 nodes with an average spacing of 9.4 mm. We used the ventricular surface extracellular potentials from both the endocardial and epicardial surfaces to simulate the potential values on the ellipsoidal cap and the torso surface using FEM, as described for the CARP dataset.

#### 2.1.0.3. Utah cage dataset

The cage dataset available in EDGAR consists of measurements from our group using a perfused, isolated canine heart preparation placed inside a cylindrical cage of dimensions ~ 10 × 10 × 15 cm (600 electrodes, with average spacing of 10.7 mm) within a human torso-shaped electrolytic tank (dimensions ~ 36 × 22 × 40 cm) instrumented with 192 surface electrodes (average spacing of 40 mm MacLeod et al., 1995; Shome and MacLeod, 2007; Milanic et al., 2014). For this study, we used recorded signals from three activation profiles: sinus rhythm and left and right ventricular pacing. The geometric model and measured potentials are all available on the EDGAR database. We used the cage electrodes as a pericardial source and compared forward computed and measured torso-tank surface potentials. We also generated simulated ground truth torso potentials from the recorded cage potentials using FEM, just as for the other two datasets.

### 2.2. Sampling strategies

**Figure 1:**
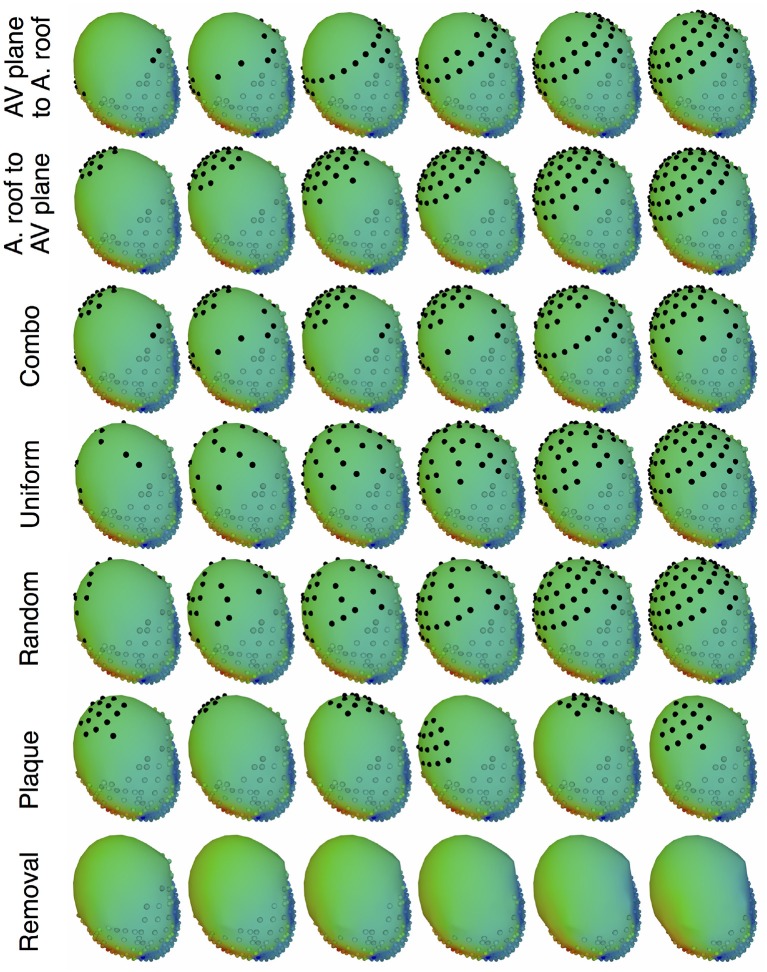
Cardiac source sampling strategies tested. Recording locations were added from the AV plane of the heart to the atrial roof, from the roof to the AV plane, a combination of the first two, uniform sampling, and random sampling of the atria. Black spheres indicate added atrial sampling locations.

The main goal of the study was to evaluate the effect of source representation in the forward solution by varying coverage and sampling density of the signals representing that source. We used five different incremental sampling strategies with each of the datasets to analyze the specific effect of atrial sampling on the simulated ECG, as shown in Figure 1. Sampling locations were added to the atria in an increasing fashion: (1) starting near the atrioventricular (AV) plane (closest to the ventricular sock) and moving toward the atrial roof, (2) from the atrial roof to the AV plane, (3) combining sites from the AV plane and atrial roof, (4) adding sites in a uniformly distributed order, and (5) adding sites in a randomly distributed order. The sampling locations were added in nine iterations for the KIT dataset, seven for the CARP dataset, and seven for the cage dataset.

In addition to testing a variable number of added electrodes to the atria, we also tested the effect of adding a cluster of electrodes, similar to a plaque electrode array, in a variety of different locations (Figure 1): 22 for the KIT dataset, 34 for the CARP dataset, and 72 for the cage datasets. The simulated plaque was generated by picking the nearest electrodes to each of the central locations. The number of plaque electrodes match the number of electrodes added in each iteration explained above, i.e., 11 for the KIT dataset, 15 for the CARP dataset, and 40 for the cage datasets.

In addition to testing the effect of missing atrial source samples, this study also evaluated the effect of missing ventricular source samples. To test this, source samples were incrementally removed from the basal region of the ventricles (Figure 1). Sampling locations were removed in eight iterations for the KIT dataset, six for the CARP dataset, and six for the cage datasets.

### 2.3. ECG forward simulation pipeline

To simulate the body surface potentials from pericardial surface potentials with various sampling strategies, we first interpolated values from the sampled cardiac surface mesh to the entire cardiac surface and then simulated the torso surface potentials. For the interpolation step, we used Laplacian interpolation (Oostendorp et al., 1989) to estimate the values missing due to undersampling and for the forward simulation we used the BEM, as implemented in SCIRun (Parker et al., 1997; MacLeod et al., 2004) with the Forward/Inverse toolkit (Burton et al., 2011). Similar to the simulations and experiments that provided the ground truth data, the torso was modeled as homogeneous outside the heart.

We compared simulated torso potentials with those from the ground truth data using several standard approaches. We first visually compared potential maps of the results during ventricular activation, identify similarities of the main features of activation. The quantitative comparisons that followed consisted of three standard error metrics, root mean square error (Ē), relative root mean squared error (*rRMSE*), and correlation (ρ), defined as follows:

(1) $$\begin{array}{l} Ē=\frac{\left||\Phi_{gt}-\Phi_{s}|\right|}{\sqrt{n}} \end{array}$$

(2) $$\begin{array}{l} rRMSE=\frac{\left||\Phi_{gt}-\Phi_{s}|\right|}{\left||\Phi_{gt}|\right|} \end{array}$$

(3) $$\begin{array}{l} \rho =\frac{\Phi_{gt}^{T}\Phi_{s}}{\left||\Phi_{gt}||||\Phi_{s}|\right|}, \end{array}$$

where Φ_*gt*_ is a vector of the ground truth BSPM values, Φ_*s*_ is a vector of the associated simulated BSPMs, and *n* is the number of body surface electrodes.

### 2.4. Validation experiments

With data acquired in experiments, we tested the sampling strategy of placing a regularly spaced array of electrodes on the atria to validate the prediction of our hypothesis. In an *in situ* open-chest preparation (Aras, 2015; Aras et al., 2016), we placed a cardiac sock with 247 electrodes around the ventricles and a plaque electrode array with 24 electrodes fixed to the atria on an accessible anterior epicardial region near the AV plane. With the electrodes in place, we recorded electrograms in sinus rhythm and as the heart developed ventricular tachycardia through the duration of the experiments.

Generating datasets for validation required the electrograms from the experiments be placed inside a complete geometric model of the torso. At the end of the experiments, we used a manual digitizer (Microscribe, Solution Technologies, Inc.) to capture the locations of anatomically distinct landmarks. We identified correspondance points from a previously generated geometric model of a human thorax, resulting in two meshes of the heart surfaces with a set of corresponding spatial reference points. These meshes were then registered using a combination of the RANSAC (Fischler and Bolles, 1981), Iterative closest point (ICP) (Besl and McKay, 1992), and thin plate spline techniques, followed by any necessary manual adjustments, implemented in MATLAB and SCIRun. To process the electrogram recordings, we isolated representative beats and performed baseline correction and filtering with the default settings in PFEIFER (https://www.sci.utah.edu/software/pfeifer.html; Rodenhauser et al., 2018).

The resulting registered meshes and processed cardiac surface recordings served as the input for our ECG forward simulation pipeline. The forward computations of body surface potentials also required closed surfaces, so we integrated the cardiac sock and atrial plaque meshes into an ellipsoidal cap similar to those described in section 2.1. Laplacian interpolation was then used to estimate the missing potential values on the cap. The resulting complete set of cardiac potentials was used in the ECG forward simulation pipeline, as explained in section 2.3. Torso potentials were simulated from cardiac potentials, with and without the additional plaque recordings, and compared using the metrics explained in section 2.3. We compared the resulting metrics to those from the simulated cardiac potentials described above (Figure 1).

### 2.5. Ethics

All experiments were performed with approval from the Institutional Animal Care and Use Committee at the University of Utah and conform to the Guide for the Care and Use of Laboratory Animals (National Institutes of Health publication No. 85-23).

### 2.6. Data availability

Some of the data used in this study (KIT and cage datasests) are available in the EDGAR database (http://edgar.sci.utah.edu), as previously noted. The rabbit model used in the CARP dataset was obtained from a third party, and requests for that data should be directed to the CARP software team (Deo et al., 2009). The raw data collected or generated for this study will be made available by the authors, without undue reservation, to any qualified researcher.

## 3. Results

Removing potentials from the atrial region of the cardiac surface had a significant impact on the computed forward simulations. For all pacing profiles and data sets, the errors in computed body-surface potentials increased when atrial samples were omitted. Furthermore, the errors grew monotonically with reduced numbers of atrial sample sites. Our experimentally recorded data also produced similar effects on the torso surface to those observed with the simulated data.

**Figure 2:**
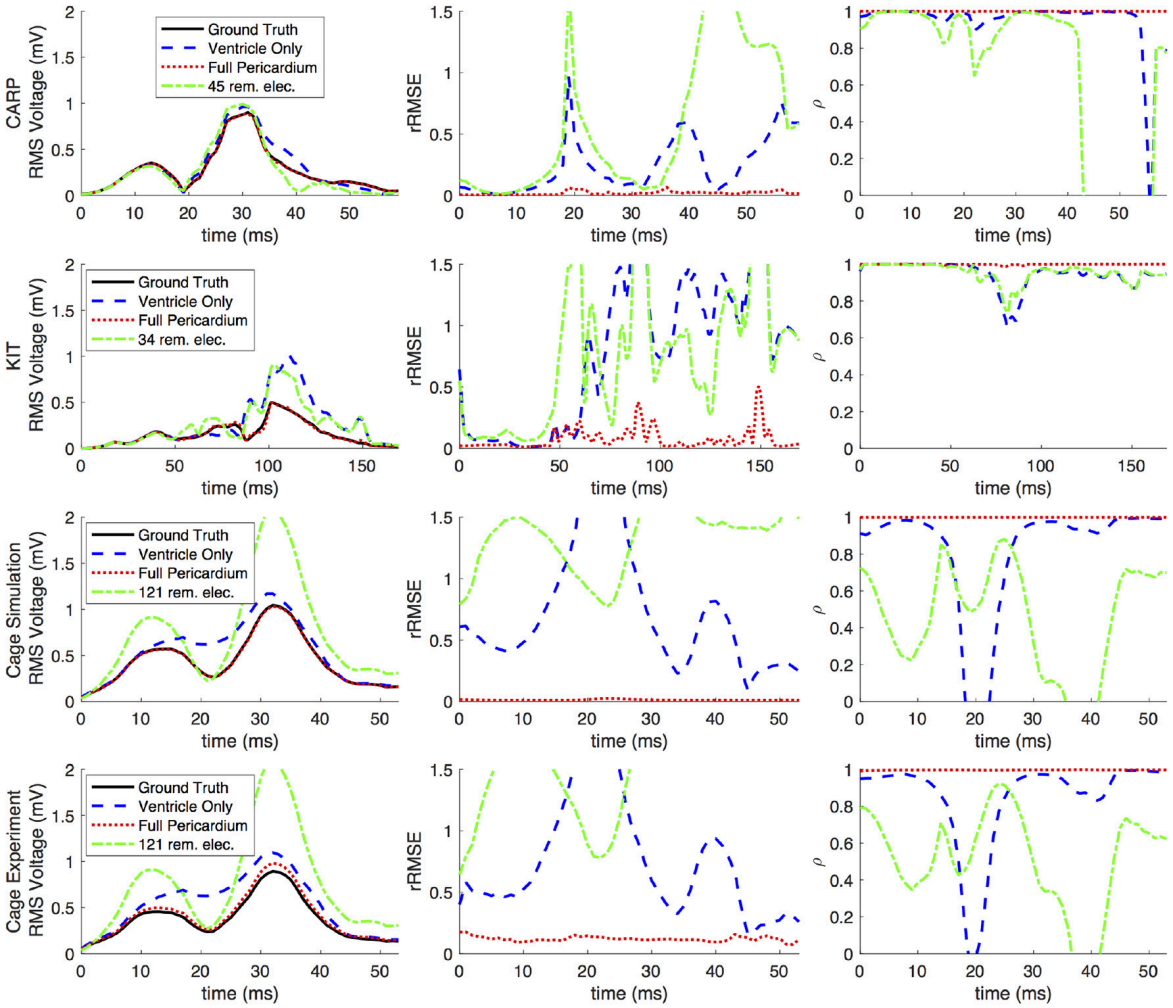
Effects of removing atrial and some ventricular sampling over time on the sinus or septal activation profile for each dataset. Each row presents the error for each dataset. Each column corresponds to a metric, RMS voltage, relative RMS error (*rRMSE*), and correlation (ρ). Each plot shows a tracing of the error over the ventricular activation in four case: ground truth (RMS voltage only), using ventricle-only sources, full pericardial sources, and when some ventricular sources are removed from the basal region.

**Figure 3:**
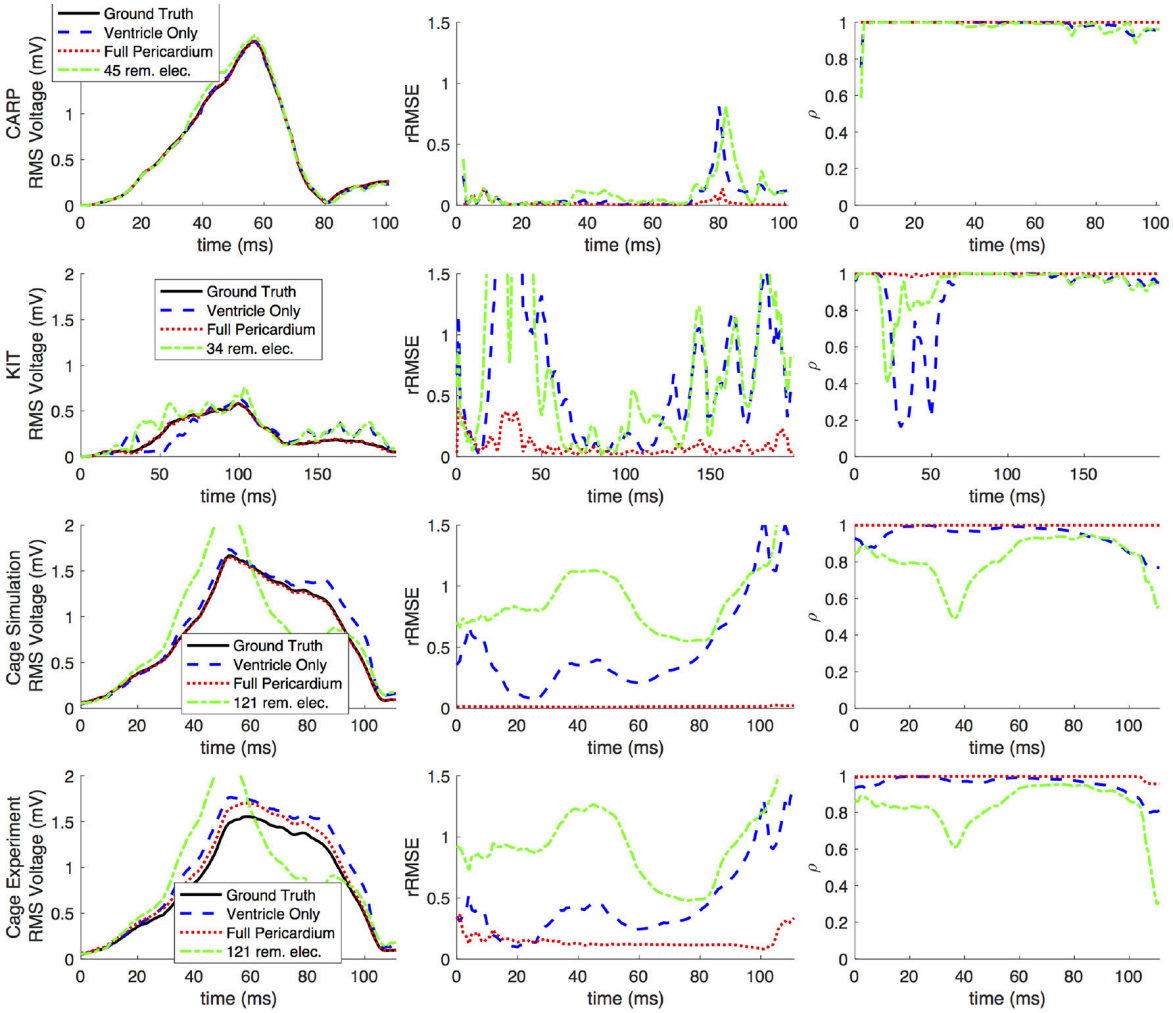
Effects of removing atrial and some ventricular sampling over time on the left ventricle simtulation activation profile for each dataset. Each row presents the error for each dataset. Each column corresponds to a metric, RMS voltage, relative RMS error (*rRMSE*), and correlation (ρ). Each plot shows a tracing of the error over the ventricular activation in four case: ground truth (RMS voltage only), using ventricle-only sources, full pericardial sources, and when some ventricular sources are removed from the basal region.

Figures 2, 3 show representative tracings of the various metrics over the course of ventricular activation with and without atrial sampling. As shown, the *rRMSE* tracings of the forward simulation using full pericardial sampling more closely match those of the ground truth. The values of ρ computed from pericardial potentials both with and without atrial sampling were high during most of the time signals, but the minima were reduced or eliminated when we included atrial sampling. The mean ρ without atrial sampling was 0.94 compared to 0.99 with atrial sampling. The *rRMSE* values showed a similar trend when comparing the forward solution with and without full atrial sampling; the maxima were reduced or eliminated when atrial samples were included. In a few time steps, adding atrial sampling produced a slight increase in *rRMSE* error, as seen in the KIT (Figure 2) and cage experiment datasets (Figure 3). However, the mean *rRMSE* was always reduced, with the total mean *rRMSE* reduced from 0.54 to 0.08. The peak Ē with only ventricular sampling ranged from 0.05 to 0.77 mV, while the peak Ē with full sampling dropped substantially, ranging from 0.01 to 0.19 mV and the peak Ē was reduced for each simulation by a mean of 0.40 mV.

**Figure 4:**
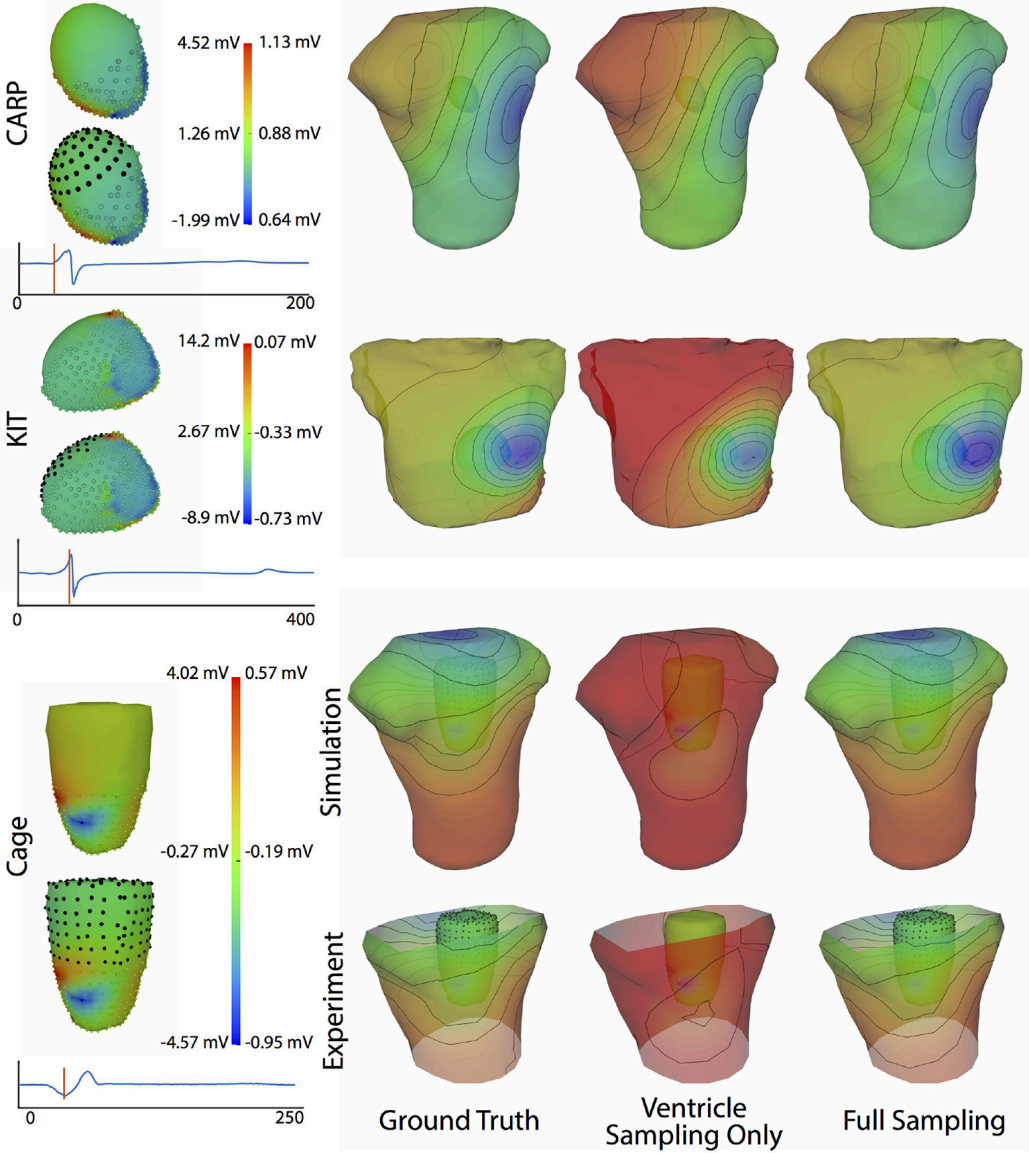
Effect of atrial region sampling on simulated BSPMs. Shown is the ground truth potential map and the forward simulation with sampling of the ventricles only and with full coverage of the ventricles and atria. The cardiac/cage surface potentials with the two sampling methods are also shown. Results are from the same representative beats shown in Figure 2 and at the time sample 25 ms into the QRS complex for the CARP dataset, 78 ms for the KIT dataset, and 18 ms for the cage datasets.

Figure 4 shows the representative cases of the general effect of excluding the potential sources in the atrial region. Comparing the potential maps simulated from only ventricular sources to the ground truth demonstrates qualitative differences, especially in the right anterior region in the CARP and KIT datasets, and over the entire anterior region with the cage datasets. However, there were no qualitative differences in the location of the extrema. The observed differences in the potential maps were reduced when we used full sampling of the atrial surface. The areas with the greatest differences were consistent across all activation profiles, as were the improvements whenever we included atrial sampling.

Increasing the number of recording locations on the atrial surface systematically resulted in reduced error in the forward simulations. Every dataset and activation profile showed a progressive decrease in the peak *rRMSE*, except the apical stimulation of the KIT dataset, which showed an increase in the peak *rRMSE* from the previous iteration when adding 22 electrodes (from 11) near the AV plane (2.85 from 1.84). The mean peak *rRMSE* over all datasets and activation profiles decreased from 2.40 to 0.06. The mean *rRMSE* also progressively decreased as atrial sampling increased in all datasets, with the same exception of the apical stimulation of the KIT dataset, which showed an increase in mean the *rRMSE* from the previous iteration (0.30 from 0.27) when adding 22 electrodes (from 11). The mean *rRMSE* decreased from 0.54 to 0.08.

**Figure 5:**
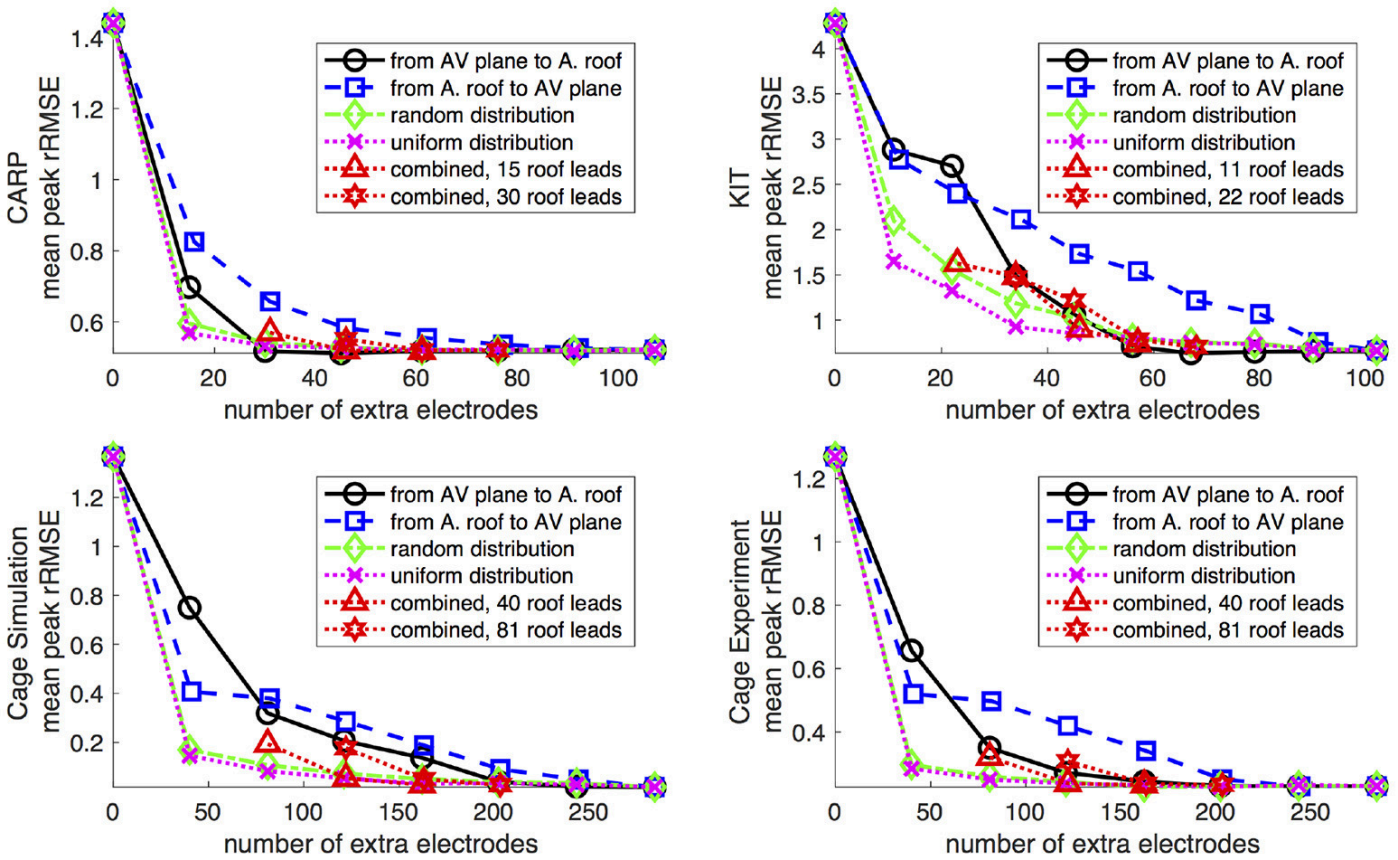
Peak *rRMSE* of the forward simulation using different sampling strategies with increasing number of electrodes. The plots are the CARP, KIT, simulated cage, and recorded cage datasets.

Figure 5 shows the mean peak *rRMSE* for each dataset. An increase in the number of samples resulted in a near asymptotic reduction in error, so that adding even a few recording locations to the atrial surface provided a significant reduction in error. Every sampling strategy we employed reduced the mean peak *rRMSE* in a similarly asymptotic relationship, but some strategies approached the minimum error with fewer added electrodes. In general, the single-direction strategies, i.e., applying electrodes only to the atrial roof or the AV plane, were less efficient than the more distributed approaches, i.e., the uniform and random distributions. The approach that combined adding electrodes to both the atrial roof and the AV plane was usually more efficient in reducing the mean peak *rRMSE* than the single-direction strategies. However, for the CARP dataset, the combined approach was only more efficient than adding electrodes to the atrial roof first. The specific order of most efficient strategies varied based on the dataset and activation profile. For example, the random distribution showed the greatest reduction of mean peak *rRMSE* after one iteration for all but the CARP dataset.

**Figure 6:**
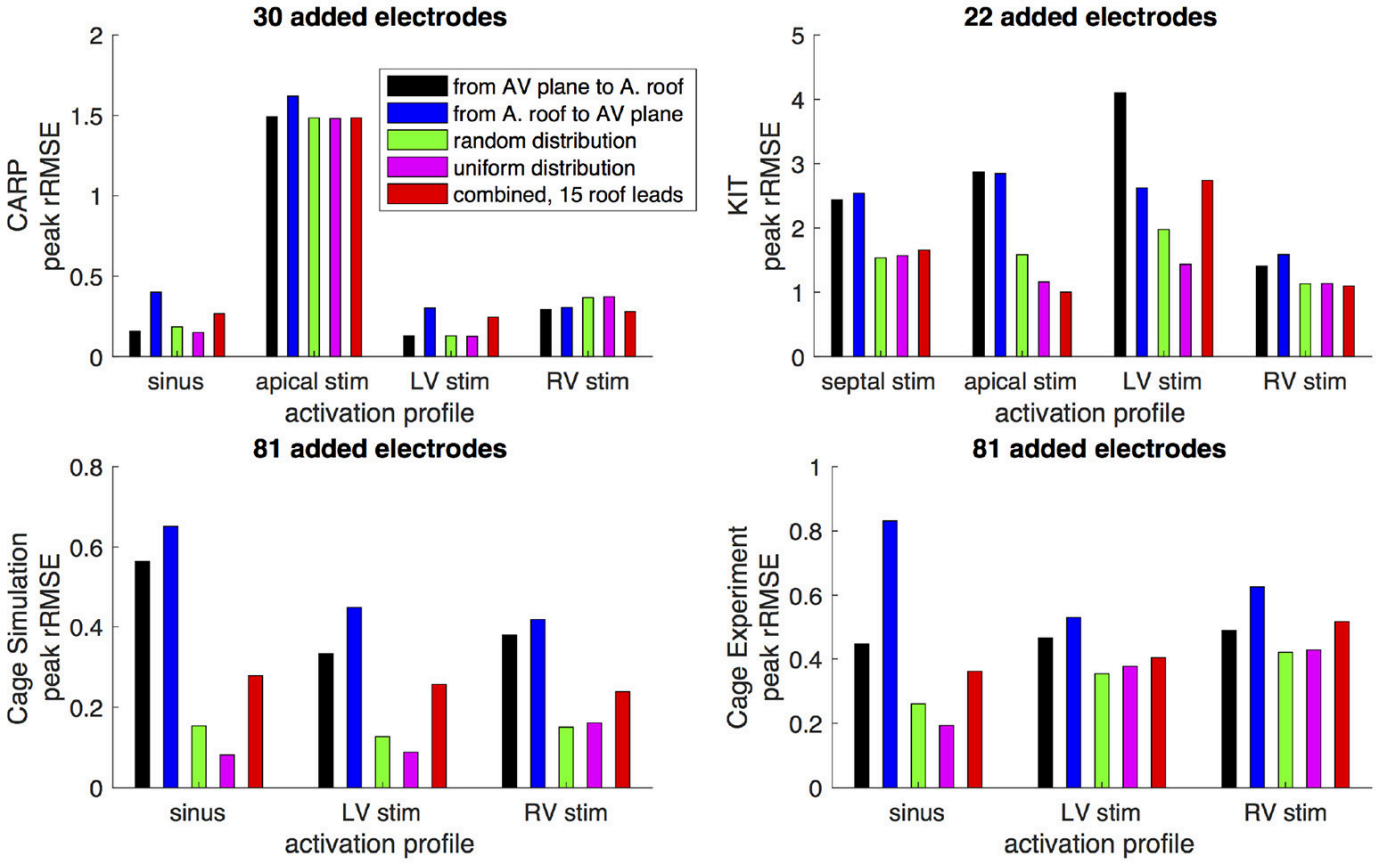
Peak *rRMSE* of the forward simulation from different activation profiles using different sampling strategies. The plots are the CARP, KIT, simulated cage, and recorded cage datasets.

**Figure 7:**
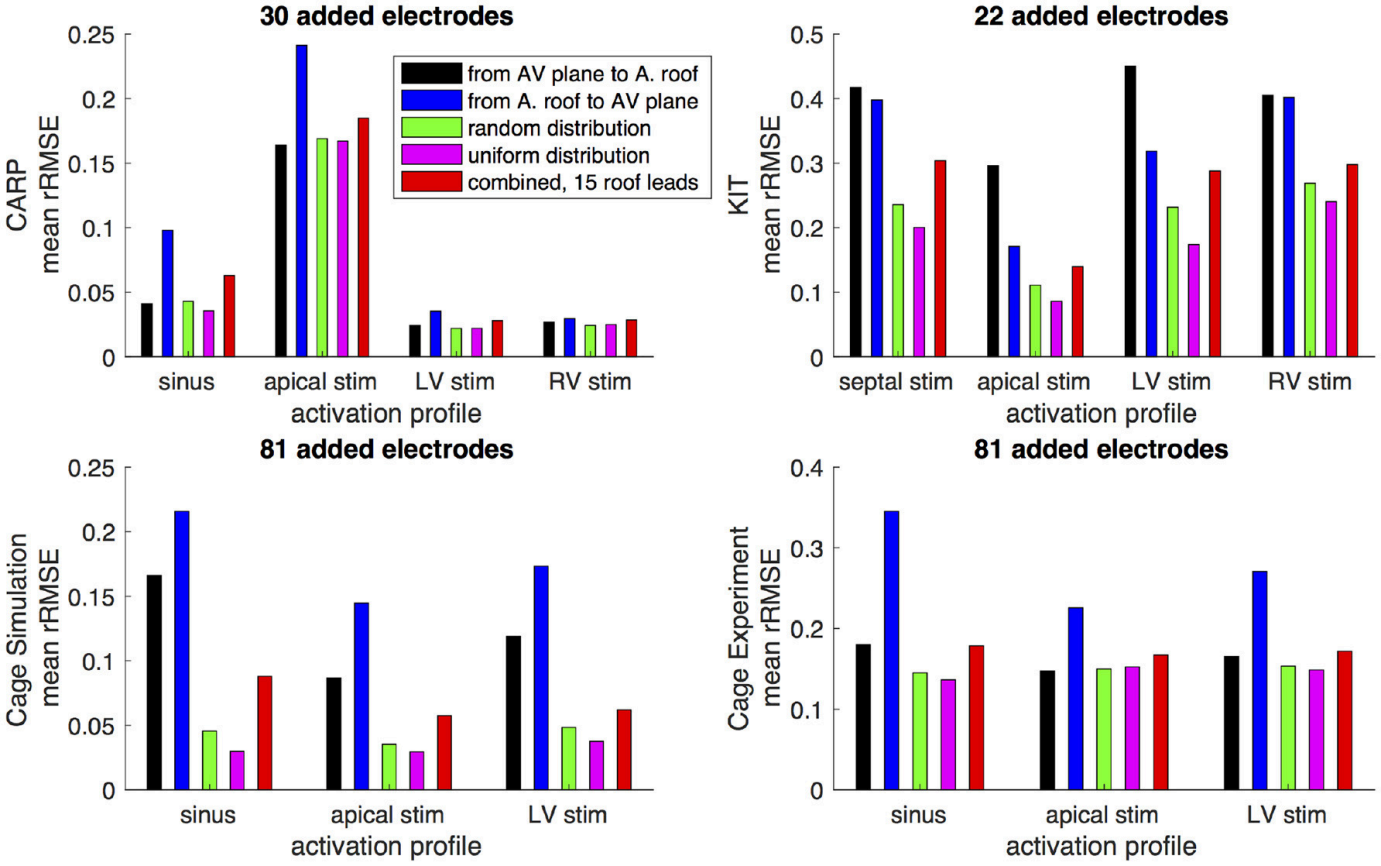
Mean *rRMSE* of the forward simulation from different activation profiles using different sampling strategies. The plots are the CARP, KIT, simulated cage, and recorded cage datasets.

Figures 6,7 show how the peak *rRMSE* and the mean *rRMSE*, respectively, were affected by the different activation profiles when adding a limited number of recording electrodes to the atria with various sampling strategies. In general, the uniform, random, and combined distributions produced lower error for each of the activation profiles than the remaining two strategies. The uniform distribution produced the lowest error of any of the strategies for most of the tested activation profiles. The random distribution had the second lowest error for most activation profiles and the combined approach was third lowest for most activation profiles. Adding recording electrodes to the atrial roof first generally had the highest error of any of sampling strategy, both in terms of the mean and peak *rRMSE*. Though there are some overall trends, there are noticeable anomalies in the responses to sampling. For instance, the apical stimulation of the CARP dataset had a noticeably higher mean and peak *rRMSE* for all sampling strategies than the other activation profiles in the same dataset. There are also cases with the CARP dataset in which the AV plane or atrial roof strategies produced lower or similar errors compared to the distributed strategies.

Simulated BSPM results from ventricular epicardial sources with potentials from an additional simulated plaque array placed in various locations showed a consistent reduction in error when compared to the simulations with ventricle-only sources. The mean *rRMSE* from all the plaque placements was 0.28 and the mean ρ was 0.97, compared to 0.40 and 0.95 with the ventricle-only sampling. The peak Ē was reduced by a mean of 0.45 mV. The placement that resulted in the lowest error was at the roof of the atria, yet there was no other trend to predict the plaque location with lower error.

When source samples were removed from the ventricular sock, there was a general increase in error for most of the QRS complex, as shown in Figure 2. By reducing the number of ventricular leads by approximately 45% of the total added on the atria (45, 34, and 121 for the CARP, KIT, and cage datasets, respectively), the mean ρ dropped from 0.94 to 0.84, the mean *rRMSE* increased from 0.16 to 0.28, and the peak Ē increased by a mean of 0.40 mV.

**Figure 8:**
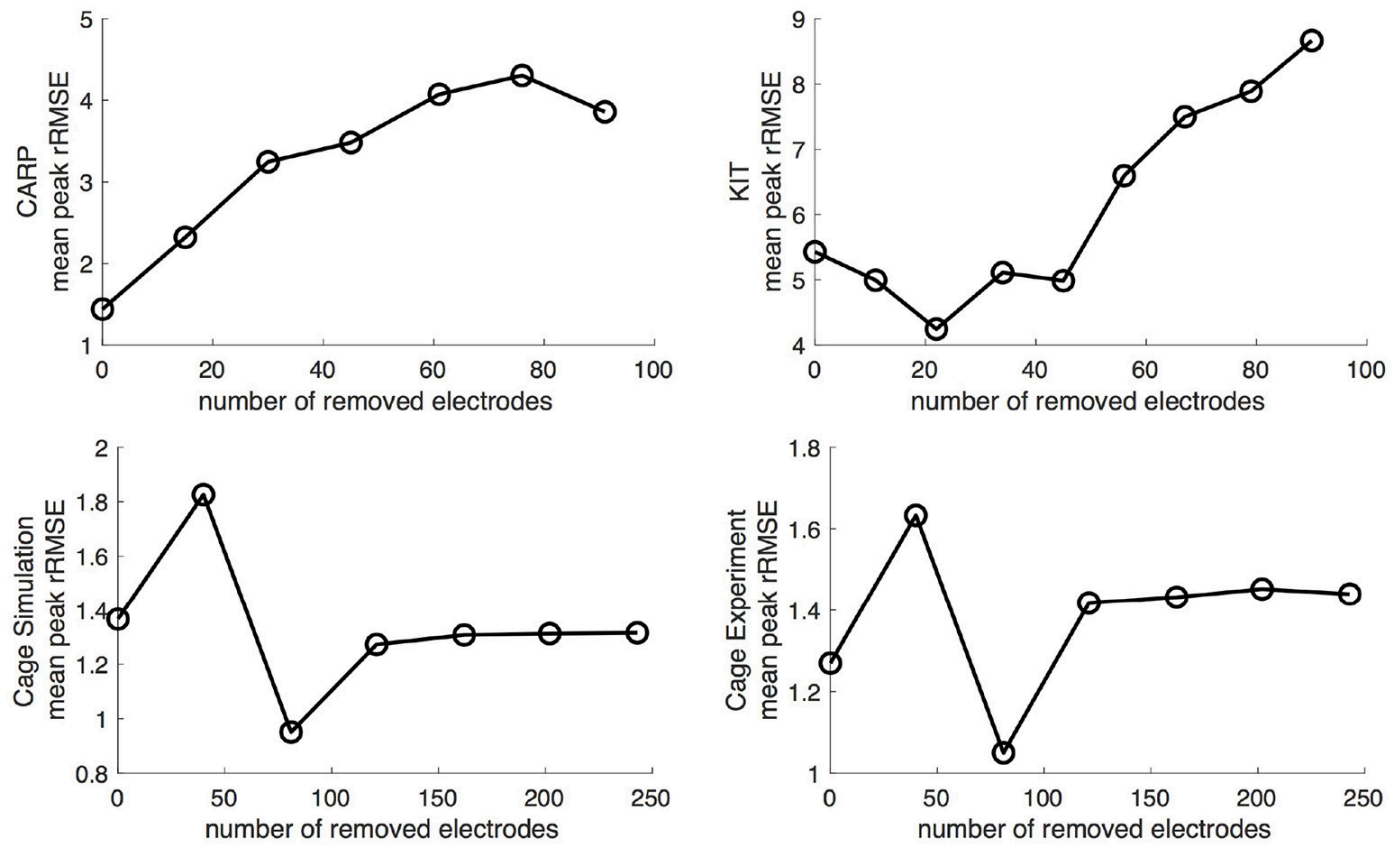
Peak *rRMSE* of the forward simulation in response to reduced ventricular sampling.

Progressively reducing the number of ventricular samples also generally increased the error, but not consistently. As shown in Figure 8, using the KIT dataset, the mean peak *rRSME* decreased initially, but then increased continuously as samples were removed. The CARP dataset showed a increased continuously as samples were removed, with the exception of the final step. Results from the cage datasets showed a similar trend: an increase in mean peak *rRMSE* with the first set of removed sources, a reduction with the second, and then a fairly consistent mean peak *rRMSE* for the remaining steps. The plateau mean peak *rRMSE* remained higher than for the full ventricular sampling for the cage experiment dataset, yet it was slightly lower for the cage simulation dataset. The mean *rRMSE* gradually increased for the CARP and cage datasets as ventricle samples were remove. However, for the KIT dataset, the mean *rRMSE* decreased slightly for the first four iterations before dramatically increase for the final stages. The mean ρ consistently dropped as samples were removed for the CARP dataset and for all but one step in the cage datasets. For the KIT dataset, the mean ρ increase slightly for three iterations, then decrease for the remaining steps.

**Figure 9:**
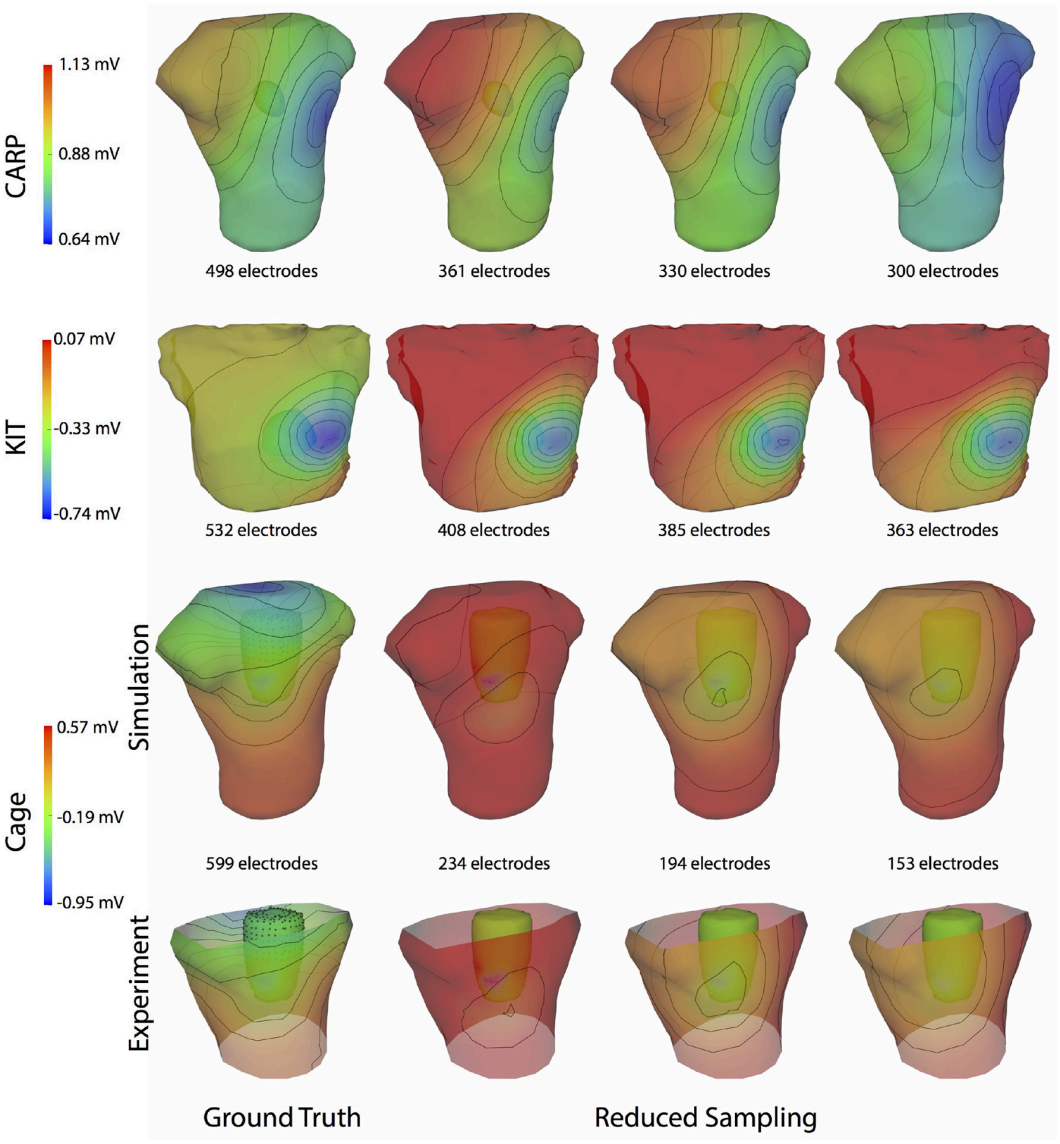
Effect of removing ventricular source sampling on simulated BSPMs. Shown is the ground truth potential map and the forward simulation with progressively reduced sampling of the ventricles. The same representative beats and time samples are shown as in Figure 4.

Figure 9 illustrates representative cases of changes in the predicted BSPMs as ventricle samples were reduced. Most notably, removing ventricular sources produced greater qualitative differences than could be generated by removing the atrial sources (Figure 4). In each dataset, removing the ventricular sources produced changes in the apparent location of the extrema on the BSPM, or, as in the case of the simulated cage dataset, removed an extremum. Interestingly, although an extremum remained missing from the BSPMs, reducing the sampling further actually otherwise improved the qualitative and quantitative accuracy of the BSPM (Figures 8, 9). This result was likely due to removing a more balanced distribution of potentials in the more extreme sampling reduction.

**Figure 10:**
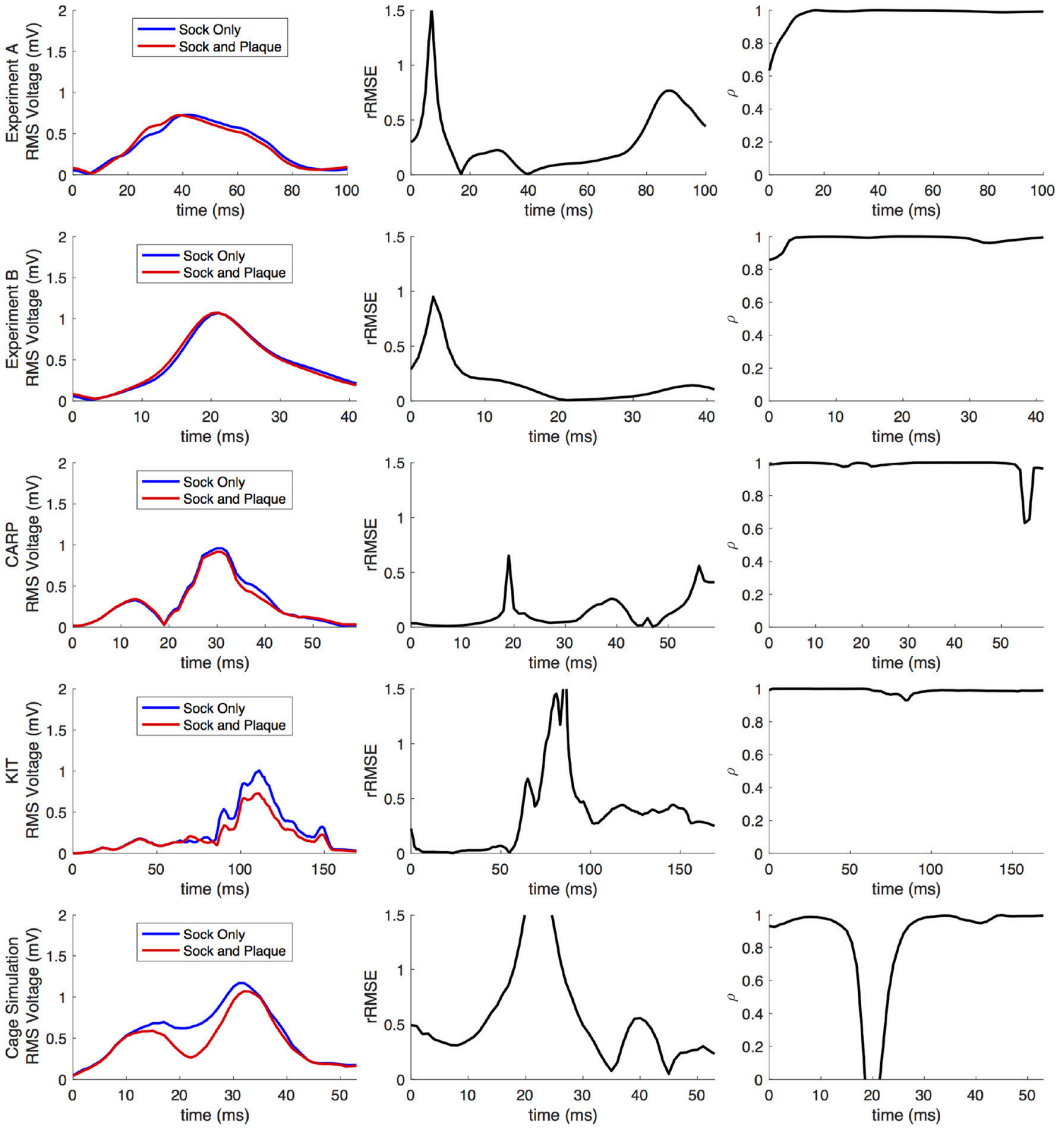
Comparison of forward simulations with cardiac sock recordings to those with additional plaque electrode recordings over time on a representative beat. Metrics from the experimental simulations and a similar comparison with simulated datasets are shown. Each row presents the error for each dataset. Each column corresponds to a metric, RMS voltage, relative RMS error (*rRMSE*), and correlation (ρ).

Comparing forward simulations using experimentally recorded cardiac sock potentials, with and without additional atrial plaque recordings, showed that the using a plaque electrode could alter the accuracy of the forward simulation. The comparison showed a mean *rRMSE* of 0.21 and a mean ρ of 0.98 across all experiments. Figure 10 shows a representative comparison over time for each of the experiments. The RMS values of the potential maps showed only minor variations, and the *rRMSE* showed some time frames with high error, most notably near the beginning of the QRS complex. The ρ remained high throughout ventricular activation, except at the beginning time instants (Figure 10, panels 1 & 2) Repeating the same experiment with simulated results yielded similar results.

Repeating the same experiment with simulated data, i.e., comparing forward simulation using cardiac sock potentials with and without an additional plaque, yielded similar results (Figure 10). The mean *rRMSE* and ρ were 0.26 and 0.98, respectively. The comparison of the BSPM over the time showed different *rRMSE* and ρ profiles compared to the experimental data, in that there peaks or dips near the middle of ventricular activation in addition to near the beginning or the end (Figure 10). However, these profiles were similar, yet with a lower amplitude, to the corresponding profiles in Figure 2 comparing the ventricle-only recordings to the ground truth data.

**Figure 11:**
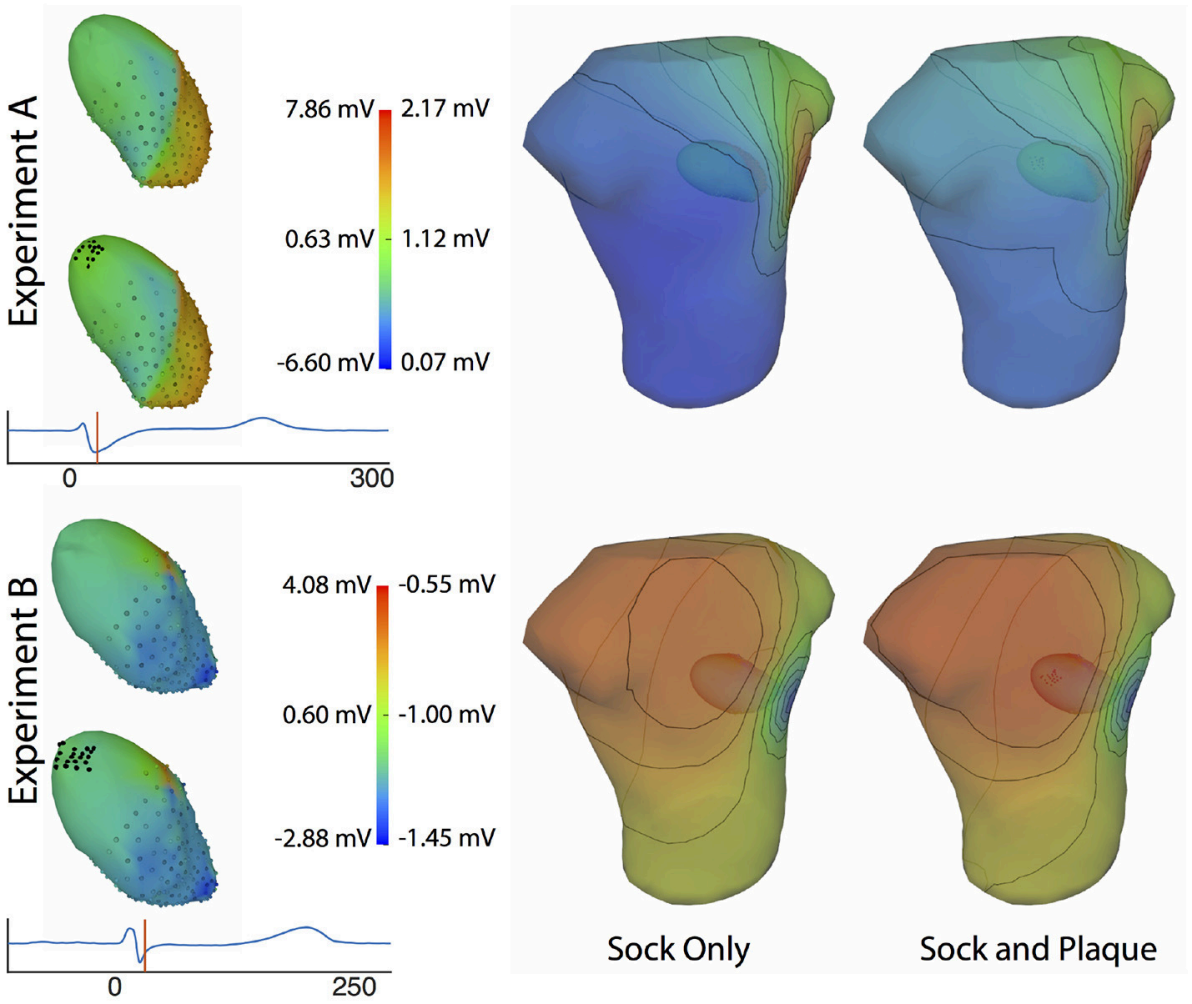
Effect of additional atrial sampling from a plaque electrode array on the forward simulation. Time frame shown is from the same representative beats shown in Figure 10 and is 30 ms into the QRS complex for Experiment A and 25 ms for the Experiment B.

Figure 11 shows the potential maps generated with and without additional recorded electrograms from a plaque based over the roof of the atria. The difference between BSPMs was relatively minor overall, but the region of greatest difference was in the right anterior region. The right posterior region also showed observable differences.

## 4. Discussion

The goal of this study was to evaluate the hypothesis that complete sampling of the cardiac surface is needed to accurately perform forward simulations of body surface potentials based on pericardial potentials, a hypothesis our results support. Moreover, our findings indicate that the accuracy of the forward simulation depends in subtle ways on the specific atrial sampling strategies. Surprisingly, some strategies are more effective than others even though they contain fewer points, indicating that sampling *location* is as important as sampling *number*. The results of this study could serve as guidance when carrying out simulations or animal and human experiments to validate electrocardiographic imaging approaches and may even impact the ECGI strategy for dealing with missing samples.

The motivation for the study came from reports and our own observations that forward simulations with ventricular pericardial sources often produced errors that exceed anticipated levels based on the relatively well-posed nature of the electrocardiographic forward problem (Ramsey et al., 1977; Bear et al., 2015). Previous, unreported results from our group based on studies with torso-tank phantoms (Shome and MacLeod, 2007) also produced a similar level of error.

The results of this study indicate that, in general, any source sampling added to the atrial region will reduce the error between the measured potentials and computed forward simulation. Even a relatively small number, *e.g*., 11–40, of additional source samples produced a reduction in the overall error (Figures 5–7) across every dataset and with every sampling strategy. Similarly, simulations that included measurements from atrial plaque electrodes also improved the agreement between ground truth torso potentials and simulations.

Although all strategies for additional atrial sampling improved the errors, we also sought specific strategies for picking the sample locations in future validation experiments. Analysis of the approaches we tested reveals that selecting evenly distributed points, such as the random and uniform strategies, are likely to produce greater accuracy with fewer added samples than other strategies (Figure 5). The combined strategy (i.e., basal plus atrial roof locations) also performed well, although not with the CARP dataset. The distributed nature of these strategies is likely a reason for their efficiency, because they reduce the need for interpolation over large distances that is an either explicit or implicit component of solving the forward problem.

Our analysis of the effect of various atrial plaque configurations on the simulated torso potentials revealed that the most valuable location may be at the roof of the atria, but placements even slightly away from the roof had lower accuracy. Therefore, it is difficult to identify and achieve the best location of additional measurement sites, typically in the form of a plaque electrode, in an experimental setting. Nevertheless, every plaque placement reduced the overall error of the simulated BSPMs, so it is likely that any plaque electrode placed on the atria will improve the overall accuracy of the forward simulation.

In comparing our results to similar studies, we found that eliminating the atrial sampling produced *rRMSE* and Ē values in the simulated torso potentials similar to those reported as early as the mid 1970s by Ramsey et al. (1977) and as recently as by Bear et al. (2015). We eliminated or dramatically reduced these errors by including sampling over the atria, which suggests that the absence of atrial sampling contributed to the errors in their studies. However, both these studies showed higher qualitative differences in simulated BSPMs, *e.g*., differences in extrema location, than we could account for by removing atrial sampling locations, which suggests additional causes of error, possibly from registration, segmentation, or addition missing sampling.

One potentially significant additional source of error is in missing ventricular sampling locations. Such undersampling of the ventricle is possible even when using a ventricular sock because parts of the epicardium may not be sufficiently sampled, for example, because of poor electrode contact around the base of the heart or a lack of electrode density in regions of high spatial complexity of the potentials. Our results indicate that eliminating sampling locations from the ventricle can produce shifts in extrema locations, or remove them entirely, (Figure 9), and, in general, will decrease the overall accuracy of the forward simulations (Figures 2, 8). Removing ventricular samples can increase the *rRMSE* even beyond that reported by Bear et al. (2015). All these results suggest that adequate sampling of both ventricles and atria is required to achieve the expected match between measured and predicted torso potentials.

The strategy of using more distributed sampling over the atria did not always produce the lowest error in the forward simulations (Figures 6, 7). The spatial variability of cardiac potentials means that there are likely sampling configurations that could reduce error more efficiently for specific geometries and activation profiles, for example, those that combined AV plane and atrial roof strategy produced the lowest error for apical stimulation in the KIT dataset, but in no other example (Figures 6, 7). Moreover, reducing error during different times of the cardiac cycle could also motivate different sampling strategies. There can be dramatic changes in the error and correlation through the cardiac cycle, as seen in the late stages of the CARP dataset sinus beat and cage datasets following left ventricular stimulation (Figures 2, 3). In a similar vein, the dramatic shift in error and correlation when the atria and basal region of the ventricles were undersampled could be attributed to incorrectly interpolating late activity near the AV plane over the atrial surface. The reduction in sampling either removes local potential extrema in this region, or could possibly remove transition regions to cause the extrema to become larger with the interpolation. In both these examples, we found that adding samples near the AV plane of the atria reduced error more than adding samples to the atrial roof (Figures 6, 7), which indicates that strategies that sample the AV plane would be important for late sinus activation or left ventricular activation. Therefore, with some *a priori* knowledge about activation profile and the regions of interest within the cardiac cycle, researchers could design specific strategies to correctly record them.

Implementing many of the strategies we tested in an experimental setting has many practical and logistical obstacles. For example, placing uniformly distributed recording electrodes on the epicardial surfaces of the atria is virtually impossible, due to limited access to the active myocardium. A combined approach including sampling near the atrial roof and near the AV plane would be feasible using multiple plaque electrodes and/or and a ventricle sock that extended over the base to the atrial surface. Such sampling would likely be feasible in an *in situ* animal preparations, although placement of the plaque would remain a challenge due to the many vessels attached to the atria. The isolated, perfused heart suspended in a torso-shaped tank phantom (MacLeod et al., 1995; Shome and MacLeod, 2007; Milanic et al., 2014), similar to the one used to acquire the cage dataset, could provide the best option for recording full coverage cardiac source potentials because the vessels supplying the heart are gathered and fed through a small opening, and the rest of the surrounding surface can be instrumented with electrodes. A limitation of this approach is that the atria are not filled with blood and so collapse to lie on the base of the ventricles and lack both realistic shape and a stable surface for attaching electrodes.

Limitations to the study generally involved compromises in capturing cardiac sources and the associated torso potentials. By using fully simulated potentials, we could achieve levels of coverage and resolution not possible with experiments but with the caveat that these are simulations and reflect certain assumptions and conditions. For example, we ignored any electrical activation of the atria, assumed that the conductivity of the atria was the same as for the torso, and greatly simplified the atrial epicardial surface by replacing it with a parameterized and smooth epicardial cap. Additionally, we did not account for possible scar or fibrosis formation which would occur in many disease states, possibly affecting any attempt to use these strategies in patients. Another source of validation data was a set of potentials from an isolated, perfused heart, captured with an instrumented rigid cage surrounding the heart. This arrangement provides full coverage of the heart and thus a complete source model, but the distance between heart and cage electrodes causes the signals to be smoother than on the epicardium and does not reflect perfectly the ECGI application. Finally, we assumed in this study that the only error would be due to insufficient source sampling of the atrial region, and thus we ignored other possible causes of error in source sampling, such as sampling density, uncertainty in individual electrode locations, or any other possible errors in capturing and representing the geometric model. These additional sources of error may compound those due to incomplete sampling over the atria.

This study focused specifically on the sampling of the atrial region and how it generally affected the forward simulation, but there are several additional, related questions that could be addressed in future studies. For example, of great interest would be a more direct spatial sensitivity analysis of the relationship between the potentials on the cardiac surface and the torso, or from the endocardial surface to the atria. Such results could suggest sampling strategies that would be specialized for specific regions of tissue, or types of activation. Other questions that could be similarly explored relate to the shape, location, and orientation of the heart, and how they might influence the forward simulation. Inclusion of torso heterogeneity due to other organs would affect the flow of current through the torso and may therefore affect the sampling strategies needed to more accurately predict BSPM. These questions and others could be the focus of future studies to help fully understand the effect of discretizing the cardiac electrical source with potential recordings.

This study illustrates the need to acquire adequate cardiac source sampling in ECG forward simulations, as well as the challenges in doing so. These findings also have implications for solving and validating the inverse solutions required for ECGI. Most mathematical formulations of ECGI solve for a subset of the cardiac sources without any cost to accuracy, but they are based on the assumption of a robust forward solution, i.e., that the relationship between the cardiac sources and the torso potentials is represented accurately (Barr et al., 1977; Plonsey and Barr, 1987; Plonsey and van Oosterom, 1991; Gulrajani, 1998). Our results suggest that coverage of the atrial surface with at least a schematic multielectrode cap could improve the resulting ECGI solutions. Additionally, our results have implications for how researchers validate ECGI methods using forward simulated BSPM data (Erem et al., 2011; Wang et al., 2011). Our findings suggest that the computed BSPMs used as inputs in these ECGI pipelines may contain errors due to inadequate cardiac sampling. Using BSPMs with such errors may bias the tuning of the constraints in the ECGI inverse problem and even alter the levels of accuracy achieved.

We conclude that complete sampling of the cardiac surface potentials is required to create realistic source descriptions for validation experiments and simulations of ECGI. Ignoring or crudely interpolating over sources on the atrial surfaces or even parts of the ventricular surface will also reduce the accuracy of simulations. Researchers can mitigate these effects by ensuring that both the full ventricular epicardium and at least some locations on the atria are sampled. Even modest coverage of the atria can increase the accuracy of the resulting simulations dramatically. Distributed sampling over the atrial will likely produce the lowest error, yet may be a challenge to implement experimentally. These efforts to improve source sampling will also improve the accuracy of the ECG forward simulations, which will further clarify the aspects of ECGI that need more research and development.

## Author contributions

JT and RM originated the study idea and developed the hypothesis. JT, KG, BB, WG, BZ, JC-F, DB, and RM contributed to the study design. JT, KG, BB, WG, and BZ contributed to data collection, organization, and processing. JT, KG, and JC-F contributed to developing the simulation pipelines used in the study. JT wrote the first draft of the manuscript. JT, KG, BB, WG, BZ, JC-F, DB, and RM contributed to manuscript revision and approved the submission of the manuscript.

### Conflict of interest statement

The authors declare that the research was conducted in the absence of any commercial or financial relationships that could be construed as a potential conflict of interest.

## References

[B1] Aras K. (2015). Bioelectric Source Characterization of Acute Myocardial Ischemia. Ph.D. thesis, University of Utah.

[B2] Aras K. Burton B. Swenson D. MacLeod R. (2016). Spatial organization of acute myocardial ischemia. J. Electrocardiol. 49, 689–692. 10.1016/j.jelectrocard.2016.02.014 26947437 PMC4853261

[B3] Aras K. Good W. Tate J. Burton B. Brooks D. Coll-Font J.. (2015). Experimental data and geometric analysis repository: EDGAR. J. Electrocardiol. 48, 975–981. 10.1016/j.jelectrocard.2015.08.008 26320369 PMC4624576

[B4] Barr R. Ramsey M. Spach M. (1977). Relating epicardial to body surface potential distributions by means of transfer coefficients based on geometry measurements. IEEE Trans. Biomed. Eng. 24, 1–11. 10.1109/TBME.1977.326201 832882

[B5] Bear L. R. Cheng L. K. LeGrice I. J. Sands G. B. Lever N. A. Paterson D. J.. (2015). The forward problem of electrocardiography: is it solved? Circ. Arrhythm. Electrophysiol. 8, 677–684. 10.1161/CIRCEP.114.001573 25834182

[B6] Besl P. McKay N. (1992). A method for registration of 3-D shapes. IEEE Trans. Pat. Anal. Mach. Intell. 14, 239–256. 10.1109/34.121791

[B7] Bishop M. J. Plank G. (2011). Representing cardiac bidomain bath-loading effects by an augmented monodomain approach: application to complex ventricular models. IEEE Trans. Biomed. Eng. 58, 1066–1075. 10.1109/TBME.2010.2096425 21292591 PMC3075562

[B8] Burton B. Tate J. Erem B. Swenson D. Wang D. Brooks D. (2011). A toolkit for forward/inverse problems in electrocardiography within the scirun problem solving environment, in Proceedings of the IEEE Engineering in Medicine and Biology Society 33rd Annual International Conference. Boston, MA: IEEE, 1–4. 10.1109/IEMBS.2011.6090052 PMC3337752 22254301

[B9] Deo M. Boyle P. Plank G. Vigmond E. (2009). Arrhythmogenic mechanisms of the purkinje system during electric shocks: a modeling study. Heart Rhythm. J. 6, 1782–1789. 10.1016/j.hrthm.2009.08.023 19959130 PMC5381712

[B10] Erem B. Ghodrati A. Tadmor G. MacLeod R. Brooks D. (2011). Combining initialization and solution inverse methods for inverse electrocardiography. J. Electrocardiol. 44:e21 10.1016/j.jelectrocard.2010.12.059

[B11] Fischler M. A. and Bolles, R. C. (1981). Random sample consensus: a paradigm for model fitting with applications to image analysis and automated cartography. Commun. ACM 24, 381–395. 10.1145/358669.358692

[B12] Gulrajani R. (1998). The forward and inverse problems of electrocardiography. EMBS Mag. 17, 84–101. 10.1109/51.715491 9770610

[B13] Johnson C. (1997). Computational and numerical methods for bioelectric field problems. Crit. Rev. Biomed. Eng. 25, 1–81. 10.1615/CritRevBiomedEng.v25.i1.10 9222078

[B14] Johnson C. (2015). Chapter 43: Computational Methods and Software for Bioelectric Field Problems, Vol. 1, 4th Edn. Boca Raton, FL: CRC Press, 1–28.

[B15] Johnson C. MacLeod R. Matheson M. (1993). Computational medicine: Bioelectric field problems. IEEE Comput. 26, 59–67. 10.1109/2.237454

[B16] Loewe A. Schulze W. H. W. Jiang Y. Wilhelms M. Luik A. Dössel O.. (2015). ECG-based detection of early myocardial ischemia in a computational model: impact of additional electrodes, optimal placement, and a new feature for ST deviation. BioMed Res. Int. 2015:530352. 10.1155/2015/530352 26587538 PMC4637443

[B17] MacLeod R. Buist M. (2010). The forward problem of electrocardiography, in Comprehensive Electrocardiology, eds Macfarlane P. van Oosterom A. Pahlm O. Kligfield P. Janse M. Camm J. (London, UK: Springer Verlag), 247–298.

[B18] MacLeod R. Taccardi B. Lux R. (1995). Electrocardiographic mapping in a realistic torso tank preparation, in Proceedings of the IEEE Engineering in Medicine and Biology Society 17th Annual International Conference (Montreal, QC: IEEE Press), 245–246.

[B19] MacLeod R. Weinstein D. de St. Germain J. D. Brooks D. Johnson C. Parker S. (2004). SCIRun/BioPSE: integrated problem solving environment for bioelectric field problems and visualization, in IEEE International Symposium on Biomedical Imaging (ISBI), Arlington, VA: IEEE Press, 1–3.

[B20] Messinger-Rapport B. Rudy Y. (1986). The inverse problem in electrocardiography: a model study of the effects of geometry and conductivity parameters on the reconstruction of epicardial potentials. IEEE Trans. Biomed. Eng. 33, 667–676. 10.1109/TBME.1986.325756 3733124

[B21] Milanic M. Jazbinsek V. Macleod R. Brooks D. Hren R. (2014). Assessment of regularization techniques for electrocardiographic imaging. J. Electrocardiol. 47, 20–28. 10.1016/j.jelectrocard.2013.10.004 24369741 PMC4154607

[B22] Oostendorp T. van Oosterom A. Huiskamp G. (1989). Interpolation on a triangulated 3D surface. J. Comp. Phys. 80, 331–343. 10.1016/0021-9991(89)90103-4

[B23] Parker S. Weinstein D. Johnson C. (1997). The SCIRun computational steering software system in Modern Software Tools in Scientific Computing, eds Arge E. Bruaset A. Langtangen H. Boston, MA: Birkhauser Press, 1–40.

[B24] Plonsey R. Barr R. (1987). Mathematical modeling of electrical activity of the heart. J. Electrocardiol. 20, 219–226. 10.1016/S0022-0736(87)80019-5 3309111

[B25] Plonsey R. van Oosterom A. (1991). Implications of macroscopic source strength on cardiac cellular activation models. J. Electrocardiol. 24, 99–112. 10.1016/0022-0736(91)90001-3 2037822

[B26] Pullan A. Cheng L. K. Nash M. Brooks D. Ghodrati A. MacLeod R. (2010). The inverse problem of electrocardiography, in Comprehensive Electrocardiology, eds Macfarlane P. van Oosterom A. Pahlm O. Kligfield P. Janse M. Camm J. (London, UK: Springer Verlag), 299–344.

[B27] Ramsey M. Barr R. C. Spach M. S. (1977). Comparison of measured torso potentials with those simulated from epicardial potentials for ventricular depolarization and repolarization in the intact dog. Circulation 41, 660–672. 10.1161/01.RES.41.5.660 908112

[B28] Rodenhauser A. Good W. Zenger B. Tate J. Aras K. Burton B. (2018). Pfeifer: Preprocessing framework for electrograms intermittently fiducialized from experimental recordings. J. Open Source Softw. 3, 472 10.21105/joss.00472 PMC6152894 30259008

[B29] Rudy Y. Lindsay B. (2015). Electrocardiographic imaging of heart rhythm disorders: from bench to bedside. Card Electrophysiol. Clin. 7, 17–35. 10.1016/j.ccep.2014.11.013 25722753 PMC4337422

[B30] Schulze W. H. W. Potyagaylo D. Schimpf R. Papavassiliu T. Tülümen E. Rudic B. (2015). A simulation dataset for ECG imaging of paced beats with models for transmural, endo-and epicardial and pericardial source imaging, in First Meeting of the Consortium for EGI Imaging (Bad Herrenalp), 1.

[B31] Shome S. MacLeod R. (2007). Simultaneous high-resolution electrical imaging of endocardial, epicardial and torso-tank surfaces under varying cardiac metabolic load and coronary flow in Functional Imaging and Modeling of the Heart, Lecture Notes in Computer Science 4466 (Berlin: Springer-Verlag), 320–329.

[B32] Stanley P. Pilkington T. Morrow M. (1986). The effects of thoracic inhomogeneities on the relationship between epicardial and torso potentials. IEEE Trans. Biomed. Eng. 33, 273–284. 10.1109/TBME.1986.325711 3957380

[B33] ten Tusscher K. H. W. J. Panfilov A. V. (2006). Alternans and spiral breakup in a human ventricular tissue model. Am. J. Physiol. Heart Circ. Physiol. 291, H1088–H100. 10.1152/ajpheart.00109.2006 16565318

[B34] Vigmond E. Hughes M. Plank G. Leon L. (2003). Computational tools for modeling electrical activity in cardiac tissue. J. Electrocardiol. 36(Suppl.), 69–74. 10.1016/j.jelectrocard.2003.09.017 14716595

[B35] Vigmond E. J. Weber dos Santos R. Prassl A. J. Deo M. Plank G. (2008). Solvers for the cardiac bidomain equations. Prog. Biophys. Mol. Biol. 96, 3–18. 10.1016/j.pbiomolbio.2007.07.012 17900668 PMC2881536

[B36] Wang D. Kirby R. Johnson C. (2011). Finite-element-based discretization and regularization strategies for 3-D inverse electrocardiography. IEEE Trans. Biomed. Eng. 58, 1827–1838. 10.1109/TBME.2011.2122305 21382763 PMC3109267

